# Barrier Protection, Skin Microbiome, and Diaper Dermatitis in Infants: Current Evidence and Future Perspectives—A Narrative Review

**DOI:** 10.3390/microorganisms14102271

**Published:** 2026-10-06

**Authors:** Oznur Ozlem Ibrahimoglu, Mehmet Serhan Boduroglu, Ismail Aslan, Ertugrul Osman Bursalıoğlu, Ayse Nilhan Atsu, Ronayi Coskun

**Affiliations:** 1 Hayat Kimya R&D Center, Kocaeli 41275, Türkiye; ozlem.ibrahimoglu@hayat.com.tr (O.O.I.); serhan.boduroglu@hayat.com.tr (M.S.B.); 2 SFA R&D Co., Ltd., Teknopark İstanbul, Istanbul 34903, Türkiye; 3 ATABIO Technologies Co., Ltd., Teknopol İstanbul, Istanbul 34903, Türkiye; 4 Hamidiye Faculty of Pharmacy, Department of Pharmaceutical Technology, University of Health Sciences, Istanbul 34668, Türkiye; 5 Faculty of Pharmacy, İstanbul Kent University, Istanbul 34406, Türkiye; 6 Department of Medical Pharmacology, Faculty of Medicine, İstanbul Medeniyet University, İstanbul 34700, Türkiye; 7 Department of Medical Services and Techniques, Vocational School of Health Services, Sinop University, Sinop 57000, Türkiye; ebursalioglu@sinop.edu.tr; 8 Vocational School, İstanbul Kent University, Istanbul 34406, Türkiye; aysenilhan.atsu@kent.edu.tr; 9 Department of Pharmacy Management, Hamidiye Faculty of Pharmacy, University of Health Sciences, Istanbul 34668, Türkiye; ronayi.coskun@sbu.edu.tr

**Keywords:** diaper dermatitis, infant skin, skin barrier, emollients, microbiome modulation, diaper formulations

## Abstract

Diaper dermatitis is one of the most common inflammatory skin conditions in infancy. It develops through the combined effects of moisture, friction, irritants, and disruption of the epidermal barrier. This review aimed to evaluate recent evidence on barrier-supportive ingredients used in infant diaper care, with a focus on barrier protection, microbiome-related approaches, and their role in preventing and managing diaper dermatitis. A structured literature search of PubMed, Scopus, and Web of Science identified clinical studies involving infants and young children of diaper-wearing age that evaluated diaper-area interventions or implementation strategies and reported diaper dermatitis or related skin outcomes, including erythema, transepidermal water loss, skin pH, and hydration. The final clinical evidence synthesis included 35 independent studies reported in 34 publications. Reviews and relevant experimental or mechanistic studies were used to provide background and biological context but were not considered direct evidence of clinical efficacy in infants. Clinical findings were synthesized narratively according to intervention type, study design, population, outcomes, and key limitations. Direct clinical evidence supports barrier-protective care, appropriate cleansing practices, and diaper-related strategies as core components of diaper dermatitis prevention and management, although findings varied by formulation, population, and outcome. Evidence for botanical and natural topical interventions was formulation-specific and inconsistent across studies. Microbiome-targeted approaches, including postbiotics, are biologically plausible and supported primarily by experimental and mechanistic evidence; however, direct clinical evidence in infant diaper dermatitis remains limited.

## 1. Introduction

Diaper dermatitis is a common inflammatory skin disorder during infancy, with recent global evidence reporting incidence rates ranging from 36% to 75% across home and hospital settings and severe cases accounting for approximately 1% to 24% [1]. It typically develops in the diaper area under conditions involving moisture, friction, occlusion, and prolonged exposure to urine and feces [1,2]. Clinically, diaper dermatitis does not represent a single condition. Irritant diaper dermatitis is the most common form; however, *Candida*-associated dermatitis, allergic contact dermatitis, seborrheic dermatitis, inverse psoriasis, Jacquet erosive dermatitis, and secondary bacterial infection should be considered in the differential diagnosis. These conditions differ in their clinical features, underlying mechanisms, and management [3,4]. Accordingly, the present review focuses primarily on irritant diaper dermatitis and its prevention, while infectious and other inflammatory conditions are considered where relevant to differential diagnosis or targeted treatment.

Several factors contribute to epidermal disruption in the diaper environment. Increased skin pH and elevated enzymatic activity may impair barrier integrity and promote both irritation and microbial changes [1,3]. Depending on severity, clinical presentation may range from mild erythema to more extensive inflammatory lesions [1,3].

Both observational and clinical studies continue to highlight the importance of early skincare interventions in neonatal populations [1,5]. Perineal erythema has been reported frequently in premature infants and appears to occur more often in those with lower gestational age or frequent stool exposure [6]. In neonatal care settings, routine skincare protocols have also been associated with lower rates of diaper dermatitis and improved skin condition overall [5].

In recent years, increasing attention has been directed toward the role of the skin microbiome in maintaining epidermal homeostasis during infancy [7,8]. Shortly after birth, neonatal skin becomes colonized by diverse microbial communities that contribute to immune development and epidermal adaptation [9,10]. However, because neonatal skin remains functionally immature during early life, the balance between microbial colonization, barrier integrity, and immune activity may be more susceptible to disruption [11]. The growing interest in microbiome-oriented and skin-supportive strategies has therefore increased the need for a better understanding of their biological and clinical relevance in infant skincare [12].

The growing understanding of epidermal barrier biology and the infant skin microenvironment has increased interest in supportive diaper-care strategies [1,13]. Current formulations frequently include emollients, pH-buffering systems, botanical compounds, and microbiome-oriented approaches.

In parallel, modern diaper products are increasingly designed for absorbency, barrier integrity, and a more stable skin environment. Some newer approaches also aim to support diaper-area skin barrier function and maintain a favorable skin environment [14].

Recent advances in infant diaper-care formulations have increased the use of ingredients intended to support barrier integrity, regulate skin pH, and maintain the local skin microenvironment. At the same time, growing interest in the infant skin microbiome has led to the development of microbiome-oriented approaches, including probiotics, prebiotics, and postbiotics. However, the clinical relevance of many of these strategies remains uncertain. While some ingredients show promising mechanistic effects, clinical findings are often inconsistent and vary across studies. In addition, evidence supporting emerging microbiome-targeted interventions is still limited. Therefore, an updated evaluation of both established and emerging ingredient-based approaches is needed to better understand their roles in the prevention and management of diaper dermatitis [12,15]. Compared with the earlier review by Petek et al. [15], the present review incorporates more recent clinical and microbiome-related evidence published through 2026.

This review provides an overview of supportive skincare strategies in diaper care, focusing on their mechanistic and clinical relevance to epidermal physiology and host–microbial interactions in the diaper area.

## 2. Methods

This article is a narrative review supported by a structured literature search rather than a systematic review. A structured search was conducted in PubMed, Scopus, and Web of Science for relevant English-language publications published between 2020 and 2026. The search covered four predefined domains: clinical studies of diaper dermatitis, skin barrier function and pH, microbiome- and postbiotic-related approaches, and botanical ingredients and natural products. The search was last updated on 15 September 2026. The 2020 starting date was selected to focus on contemporary evidence; selected earlier studies of particular relevance were considered as contextual background where appropriate. The search strategies were reviewed for consistency across databases, while database-specific syntax, field tags, and search structure were retained where necessary. The complete database-specific search strategies, including the full search strings, Boolean operators, field restrictions, and the number of records retrieved from each database, are provided in Supplementary Table S1.

After deduplication across databases and search domains, 435 unique records underwent title and abstract screening. For the direct clinical evidence synthesis, no predefined numerical age range was used as an eligibility criterion; studies involving infants and young children of diaper-wearing age were considered eligible if they evaluated an intervention or implementation strategy relevant to diaper-area care and reported clinical outcomes related to diaper dermatitis or diaper-area skin. Studies reporting diaper dermatitis as a secondary clinical outcome or intervention-related diaper-area skin-barrier outcomes, such as erythema, transepidermal water loss, skin pH, or hydration, were also eligible. Non-interventional studies, including observational, microbiome-only, measurement/validation, and mechanistic studies, were excluded from the direct clinical evidence synthesis but were retained as contextual evidence where relevant.

**Figure 1:**
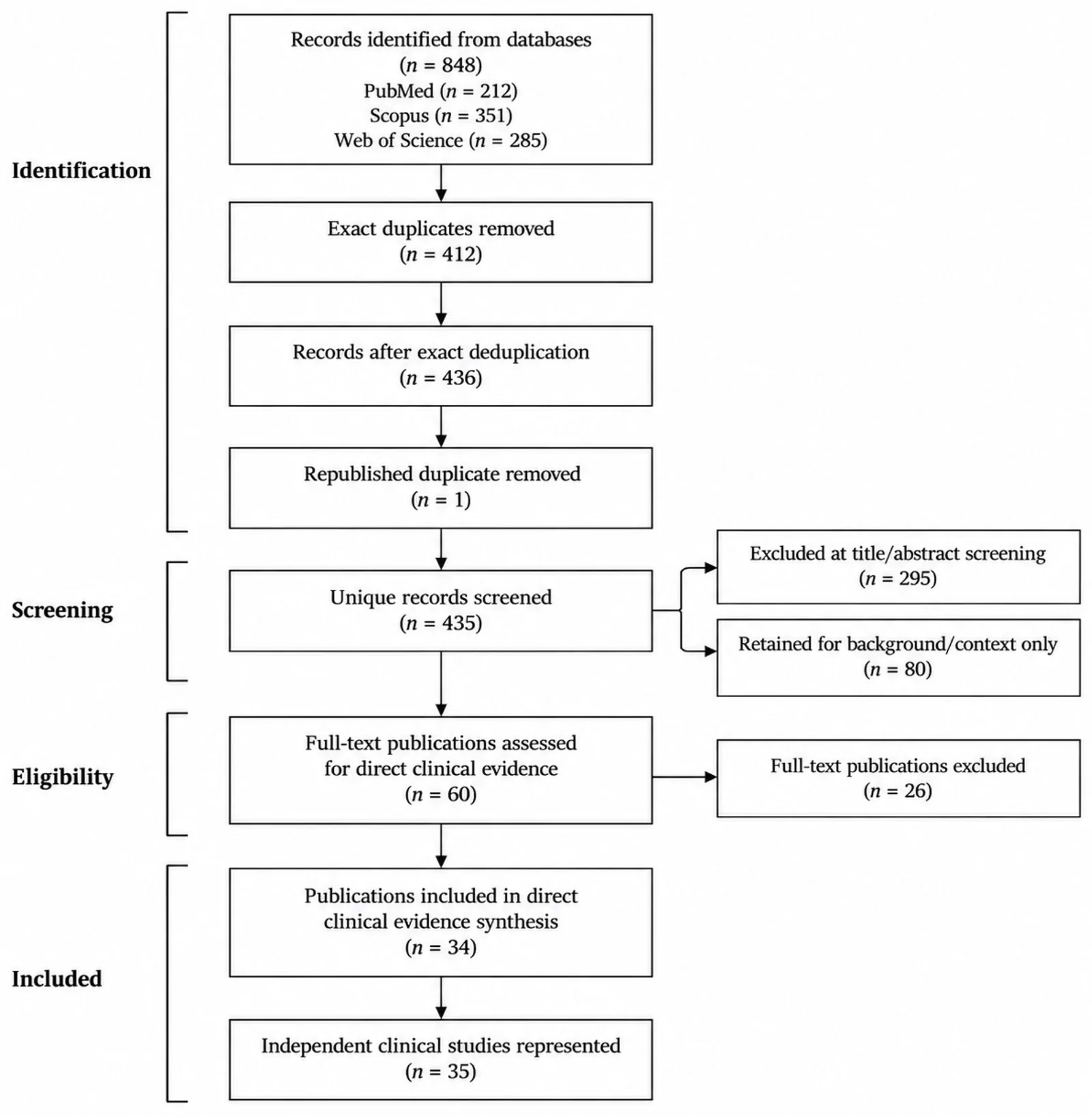
Flow diagram of study identification and selection for direct clinical evidence synthesis.

Following the updated screening and reassessment, 35 independent clinical studies reported in 34 publications were included. A total of 60 publications underwent full-text assessment for direct clinical eligibility, of which 26 were excluded from the direct clinical synthesis. The study identification and selection process is summarized in Figure 1. One publication contained two independent randomized clinical trials and was therefore represented as two studies in the clinical evidence synthesis. Study eligibility was independently assessed by two authors, and any differences regarding study selection were resolved through discussion and consensus. Reviews, consensus papers, and selected experimental or mechanistic studies relevant to skin barrier biology, microbiome interactions, formulation characteristics, or proposed mechanisms were retained separately as supporting background evidence and were not interpreted as direct evidence of clinical efficacy in infants.

The included clinical studies were synthesized narratively according to clinical purpose, intervention type, study design, population, comparator, reported clinical outcomes, safety, funding, and major limitations. Findings that differed across studies were synthesized descriptively with consideration of differences in populations, interventions, comparators, outcomes, and study settings. Differences in diaper dermatitis severity instruments were considered as an additional source of outcome heterogeneity. Reick et al. identified only three standardized instruments and found insufficient evidence to recommend a specific diaper dermatitis severity instrument [16]. Given the narrative-review design and the substantial heterogeneity of the included studies in terms of study design, populations, interventions, comparators, and outcome measures, no formal risk-of-bias or certainty-of-evidence assessment was performed, and no formal evidence grades were assigned. Instead, major study-level methodological limitations were considered in the narrative interpretation of the clinical evidence. The absence of a formal risk-of-bias assessment limits the ability to systematically compare methodological quality across studies and should therefore be considered when interpreting the strength of the clinical findings.

## 3. Pathophysiological Basis of Diaper Dermatitis

Conditions in the diaper area, such as occlusion and excess hydration, create a sensitive environment that promotes irritation and inflammatory skin changes [17,18]. In infants, the skin barrier is still developing, which may explain their increased vulnerability to environmental and mechanical challenges within the diaper area. As epidermal disruption progresses, changes in the stratum corneum appear to play a central role in the development of diaper dermatitis [15,17].

Since epidermal maturation is not yet complete in preterm and low-birthweight infants, barrier-related vulnerabilities are more common in these populations. In addition, the relatively high surface area-to-body weight ratio in premature infants may increase exposure to topical substances, making careful formulation selection particularly important in neonatal skincare. Immature neonatal skin may respond differently to topical products than fully developed skin [17,19].

Premature infants can exhibit increased transepidermal water loss, reflecting delayed maturation of epidermal barrier function. Skin pH in preterm infants varies with postnatal maturation and anatomical site, while barrier development is also influenced by gestational age; therefore, skin pH findings should not be generalized across all premature infants [17,19]. This distinction is particularly relevant in the diaper area, where the course of pH and barrier maturation may differ from that of other anatomical sites.

Together, these features provide the mechanistic basis for strategies aimed at preserving epidermal barrier integrity; specific barrier-supportive ingredients and formulations are discussed in Section 4.

Nevertheless, the diaper environment still exposes infant skin to multiple irritants that can compromise epidermal integrity [18]. Prolonged exposure to urine and feces, increased skin pH, and elevated protease and lipase activity have all been implicated in barrier disruption [18,20]. A key mechanistic link between microbial activity and barrier disruption involves bacterial urease-mediated hydrolysis of urinary urea to ammonia. The resulting increase in local skin pH can enhance the activity of fecal proteases and lipases, thereby promoting stratum corneum damage and further barrier impairment [21]. Among these factors, alterations in skin pH appear particularly important. Disruption of the normal acidic skin environment may adversely affect epidermal barrier function [22]. In infant diaper-area skin, inflammatory changes have been associated with increased inflammatory mediator activity [22]. Separately, an experimental mouse model using synthetic feces demonstrated increases in IL-1α, IL-6, and TNF-α, supporting a mechanistic link between irritant exposure and cutaneous inflammation [20].

Changes in the diaper-area microbiome have also been associated with diaper dermatitis, skin pH, and rash severity [13,23]. However, these associations do not establish a unidirectional causal relationship. Microbiome alterations may occur alongside changes in pH, barrier integrity, inflammation, and local skincare practices, and the direction of these relationships remains uncertain.

**Figure 2:**
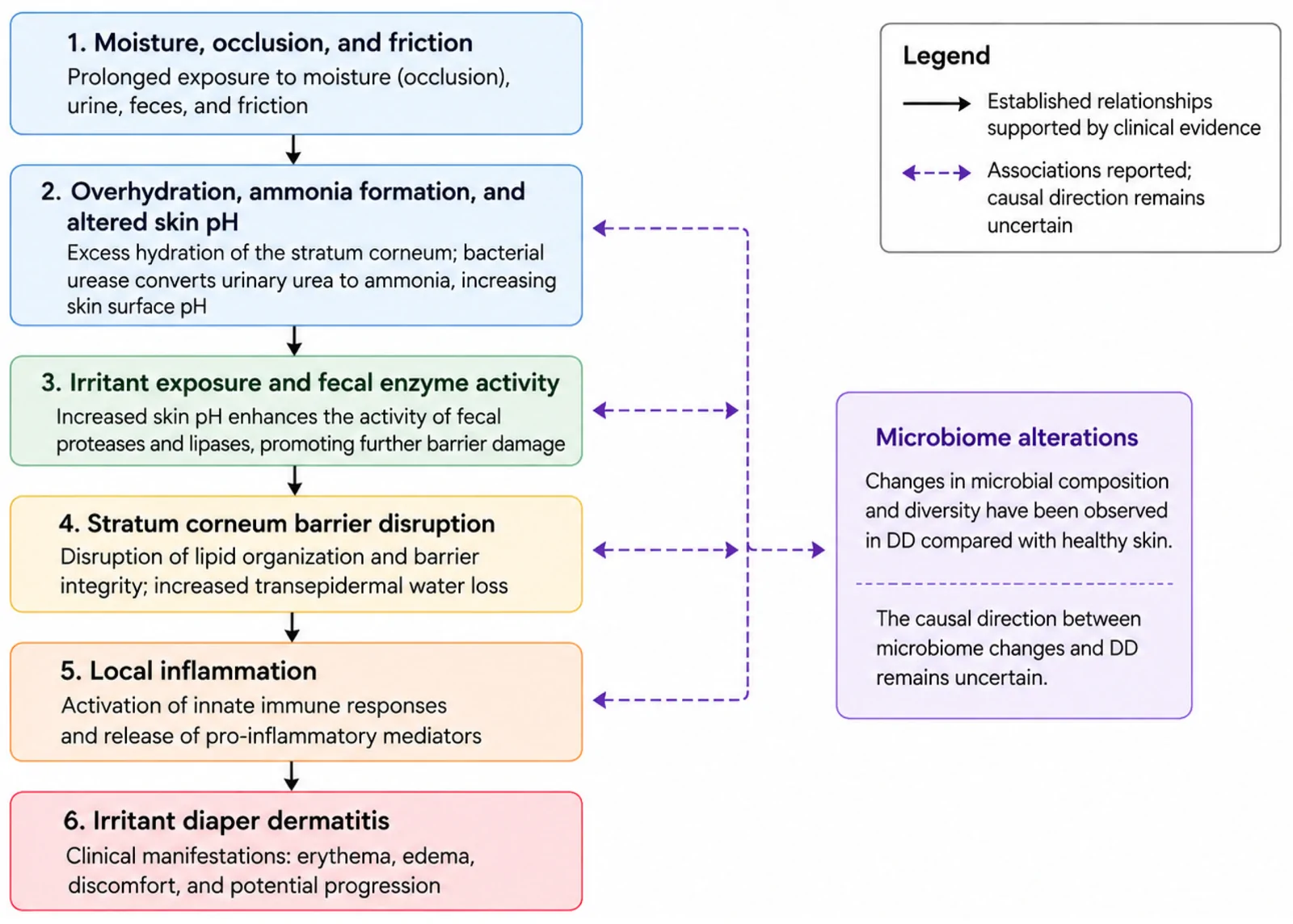
Pathophysiological mechanisms involved in irritant diaper dermatitis. Moisture, occlusion, altered skin pH, friction, and exposure to urine, feces, and fecal enzymes contribute to epidermal barrier disruption and local inflammation. Microbiome alterations may occur alongside these changes, although the direction of these associations remains uncertain.

The pathophysiology of diaper dermatitis suggests that barrier impairment is both an initiating event and a consequence of ongoing inflammation in the diaper area. Because multiple pathogenic mechanisms act simultaneously, strategies for maintaining diaper-area skin integrity may need to address several interacting factors rather than a single pathway. These interacting mechanisms are summarized in Figure 2.

## 4. Ingredients and Formulation Strategies for Diaper-Area Skin Care

Advances in infant skincare have focused on topical strategies to preserve epidermal integrity and reduce irritation in the diaper area [17,24]. Current diaper-care formulations include a wide variety of ingredients; however, the strength of clinical evidence varies substantially across ingredient categories [2,24].

Diaper-area barrier products encompass diverse formulations containing ingredients such as petrolatum, zinc oxide, lanolin, dimethicone, and dexpanthenol [18,25], with the concentration and combination of these components varying among products. Cleansing wipes likewise differ in formulation characteristics, including pH, preservative systems, and fragrance [25,26] Accordingly, clinical evidence should be interpreted at the formulation level rather than generalized solely on the basis of an individual ingredient or product category.

Emollient- and barrier-supportive approaches are commonly used in diaper care; however, the strength of clinical evidence varies according to the specific formulation, study population, and clinical outcome evaluated [18,24].

A recent systematic review and meta-analysis including six studies reported a pooled odds ratio of 0.77 (95% CI 0.37–1.61) for diaper dermatitis with substantial heterogeneity (I^2^ = 79%) [24]. The wide confidence interval and high heterogeneity indicate that the pooled estimate should be interpreted cautiously and should not be taken as evidence of a uniform benefit across different barrier preparations. The supporting evidence for botanical compounds and microbiome-oriented interventions continues to evolve [12,27].

### 4.1. Emollients, Barrier Products, pH Regulation, and Effects on Skin Barrier Function

Emollients are a cornerstone of barrier-supportive infant diaper care, as they help protect the skin barrier during prolonged occlusion, excess moisture, and constant exposure to irritants. Their main benefits include reducing transepidermal water loss (TEWL) and maintaining stratum corneum hydration. While direct mechanistic evidence specific to diaper use is limited, research on neonatal skin shows that the immature epidermis is more permeable and sensitive than adult skin, supporting early barrier-enhancing strategies [17,19].

Emollients, occlusive barrier ointments, and zinc-containing preparations are all used to protect diaper-area skin, although they act in somewhat different ways. Emollients help maintain hydration and support the lipid organization of the stratum corneum, whereas occlusive preparations limit direct contact with moisture and irritants. Zinc-containing preparations add a physical protective layer and are commonly used in diaper dermatitis care. The overall effect of these products depends on the combination of ingredients and formulation characteristics rather than on a single component alone [24,28].

The composition of a formulation can also affect its impact on the skin barrier. Lipid-supportive formulations have been associated with improvements in TEWL and other measures of barrier function, although the magnitude of these effects differs across formulations and study populations [28,29]. Changes in visible dryness may not always parallel changes in objective measures of barrier function, and improvement in clinical appearance should therefore not be assumed to represent complete barrier recovery [29].

Skin pH is another factor relevant to barrier function in the diaper area. Maintenance of an acidic skin surface supports epidermal homeostasis and processes involved in barrier integrity [17,19]. pH is also associated with the local microbial environment, but these relationships are complex and do not demonstrate that pH-related changes in the microbiome directly protect against diaper dermatitis [13,23]. Some diaper-care regimens therefore combine barrier-supportive products with pH-buffering approaches, with clinical studies reporting effects on outcomes such as skin pH, erythema, and residual enzymatic activity [30,31].

### 4.2. Botanical Ingredients and Natural Topical Agents

Botanical ingredients have long been incorporated into infant skincare for their potential anti-inflammatory, antioxidant, and skin-soothing properties [32,33,34,35]. Among the plant-derived oils used in infant and pediatric care are almond, olive, and chamomile oils, which have been used in traditional and clinical skincare for infants and children [27,33,34].

The biological effects of botanical ingredients may involve inflammatory pathways and the skin microenvironment [32,33,35]. Although these ingredients remain widely used in infant skincare, clinical evidence varies according to the specific botanical preparation and study design [27,36,37,38].

Moreover, botanical extracts such as *Aloe vera*, *Calendula officinalis*, and *Matricaria chamomilla* are commonly incorporated into diaper-care formulations and other topical products used in the diaper area [27,32,34]. However, evidence supporting natural topical interventions remains inconsistent. Community-based studies, randomized trials, and systematic reviews of plant-based oils and emollients have reported mixed or limited benefits for diaper dermatitis, atopic dermatitis, infections, and other neonatal outcomes, with considerable methodological heterogeneity across studies [27,36,37,39,40,41]. Certain oil-based approaches have also been associated with adverse effects on barrier parameters, including increased transepidermal water loss under specific conditions [36,38]. Overall, the clinical effectiveness of botanical and oil-based preventive strategies in infant skincare remains uncertain and may depend on formulation characteristics, application practices, and developmental differences in infant skin physiology [28,36,38].

Differences in formulation composition can affect hydration, TEWL, lipid lamella structure, and microbiological stability, thereby altering barrier effects in infants [34,39].

Application practices such as massage frequency, localized use, and controlled environments also influence skin responses and help explain the divergent results seen between hospital-based and community-based studies [38].

Studies on neonatal and infant skin barrier biology emphasize that the immature, thinner, and more permeable epidermis in preterm infants modulates how oils interact with the barrier, suggesting that developmental stage and underlying risk profile are important modifiers of outcomes [17,19,38].

### 4.3. Microbiome-Modulating Approaches

Interpretation of diaper-area microbiome findings requires consideration of both biological and methodological factors. Relative changes in individual taxa do not necessarily indicate dysbiosis or functional microbial disturbance without supporting quantitative or functional evidence. Microbiome composition in early life may also be influenced by age, mode of delivery, feeding, antibiotic exposure, environmental factors, and methodological differences in sampling and sequencing [8,9,10,11]. Relative abundance data do not necessarily reflect absolute microbial load. Therefore, microbiome findings in diaper dermatitis should primarily be interpreted as compositional associations unless functional or quantitative alterations have been demonstrated.

Earlier primary evidence also suggested an association between diaper dermatitis and alterations in the diaper-area skin microbiome. Zheng et al. (2019) [42], published before the predefined search period of the present review, reported microbiome differences in diaper dermatitis lesions and changes during recovery following emollient treatment. Collectively, these observations have increased interest in strategies that extend beyond conventional antimicrobials, including microbiome-oriented approaches to diaper-related care [12,15].

In diaper-area microbiome studies, *Staphylococcus epidermidis* has been identified as part of the normal skin microbiota, whereas *Staphylococcus aureus* and fecal-associated *Enterococcus* have shown higher relative abundance in more severe rash states [13,23]. Among preterm infants, Younge et al. reported no significant difference in diapered-skin alpha diversity between 3-h and 6-h diaper-change groups, while diaper-change frequency explained only a small proportion of overall variation in community structure [43]. The functional significance and causal direction of these compositional changes remain uncertain. Fungal involvement should also be considered separately, particularly in *Candida*-associated diaper dermatitis. *Candida albicans* has been associated with affected diaper-area skin and may become more prominent under moist and occlusive conditions, particularly when barrier integrity is impaired [3,15,23]. However, *Candida*-associated dermatitis represents a distinct clinical presentation and should not be considered equivalent to uncomplicated irritant diaper dermatitis.

Table 1 summarizes the available primary evidence characterizing the infant diaper-area microbiome, with emphasis on study population, sampling methodology, microbial analysis, key findings, and methodological limitations.

Early-life microbial development is also influenced by host and environmental factors. Breastfeeding, delivery mode, hygiene practices, topical products, and other environmental exposures have been associated with differences in the developing infant skin microbiome [8,9,10,11]. Feeding practices also influence intestinal microbial development, raising interest in possible interactions between the gut microbiota, immune development, and skin homeostasis through the gut–skin axis. However, direct evidence linking these systemic interactions to infant diaper dermatitis remains limited, and findings from intestinal or other dermatological conditions should not be extrapolated directly to diaper dermatitis [10,11,12].

#### 4.3.1. Conventional Antimicrobial Approaches

Conventional antimicrobials may be used when bacterial or fungal involvement is clinically suspected or confirmed in the diaper area [3,18,44]. However, topical antibacterial and antifungal agents should not be considered routine components of daily prevention of uncomplicated irritant diaper dermatitis. *Candida*-associated or bacterial complications should be distinguished from uncomplicated irritant diaper dermatitis and treated according to the clinical indication [3,18].

Unnecessary or prolonged use of topical antimicrobial agents may alter resident microbial communities, contribute to local irritation, and, particularly for antibacterial agents, increase selective pressure for antimicrobial resistance [44,45]. Therefore, antimicrobial treatment should be targeted to suspected or confirmed infectious involvement rather than used universally for prevention.

Together, these findings support a distinction between routine barrier-supportive preventive care and targeted antimicrobial treatment when clinically indicated.

#### 4.3.2. Direct Clinical Evidence for Microbiome-Targeted Approaches

Clinical evidence specifically addressing microbiome-targeted interventions in infant diaper dermatitis remains limited. Available clinical studies have mainly evaluated probiotic approaches administered through routes other than topical diaper-area application [12,46]. For example, Dimitratos et al. reported caregiver-observed changes in diaper rash following oral administration of *Bifidobacterium longum* subsp. *infantis* EVC001; however, the study was uncontrolled and observational, and the intervention was not applied topically [46]. These findings therefore cannot establish the efficacy of topical probiotics or postbiotics for the prevention or treatment of diaper dermatitis.

At present, direct controlled clinical evidence for topical postbiotic formulations in infant diaper dermatitis is lacking [12,15]. Further clinical studies are needed to determine their efficacy, safety, appropriate formulation, and potential role in diaper-area care.

#### 4.3.3. Indirect and Preclinical Evidence for Microbiome-Targeted Approaches

Probiotic, prebiotic, and postbiotic approaches have been investigated for their potential effects on microbial communities, barrier function, and local immune responses in experimental and skin-related models [12,15,47,48]. According to the International Scientific Association for Probiotics and Prebiotics (ISAPP), a postbiotic is defined as “a preparation of inanimate microorganisms and/or their components that confers a health benefit on the host.” Postbiotics are distinct from probiotics, which contain live microorganisms, and prebiotics, which are substrates selectively utilized by host microorganisms [49]. Advances in formulation and diaper-related technologies have also led to experimental investigation of postbiotic-containing topical formulations and functional materials [12,48,50].

Experimental studies have evaluated specific postbiotic preparations in skin-related models. A *Latilactobacillus curvatus* BGMK2-41-derived postbiotic showed barrier-related and antimicrobial effects in keratinocyte and ex vivo human skin models [48], while a separate postbiotic complex demonstrated antibacterial and anti-inflammatory activity in experimental skin models [47]. In a previous study by members of our research group, postbiotic-containing materials were also explored in prototype diaper-related systems [50]. However, none of these studies directly evaluated infant diaper dermatitis, and their findings should therefore be considered preclinical rather than evidence of clinical efficacy or safety.

Postbiotics are non-viable microbial preparations and may offer practical formulation advantages, including greater stability during processing and storage compared with live microorganisms [12,49]. However, the absence of viable microorganisms does not by itself establish safety. Depending on their composition and manufacturing process, postbiotic preparations may contain cellular components, metabolites, residual nucleic acids, allergens, or other biologically active molecules that require appropriate characterization and toxicological assessment [49,51]. Therefore, formulation-specific characterization and clinical safety assessment are required, particularly for use on infant skin. Their potential application in diaper care remains an area for further investigation [12].

## 5. Clinical Management and Preventive Care Strategies

### 5.1. First-Line Clinical Care and Preventive Strategies

Clinically, the prevention and management of diaper dermatitis center on topical interventions and daily care practices [1,2,3,18,24]. The clinical purpose of the included interventions varied across studies: some evaluated prevention of diaper dermatitis or maintenance of diaper-area skin integrity, whereas others evaluated treatment or resolution of established dermatitis. These purposes are distinguished in the study-level evidence summary to avoid interpreting preventive and therapeutic outcomes as equivalent. Diaper-change frequency represents another component of preventive care, particularly in preterm infants, in whom both prolonged exposure to the diaper environment and repeated handling may be clinically relevant. Randomized evidence evaluating different diaper-change frequencies has considered skin health alongside physiological stability, supporting the need to balance diaper-area care with the vulnerability of preterm infants to frequent caregiving interventions [52].

Routines that include emollient-containing products and pH-balanced cleansing have been linked to reduced erythema and maintenance of physiological skin pH in the diaper area [14,30,31]. Moisturizer application has also been evaluated in term newborns as a strategy for supporting perianal skin-barrier function, providing evidence for barrier maintenance rather than treatment of established diaper dermatitis [53]. However, the effectiveness of these approaches varies by product composition, frequency of use, and baseline skin condition.

Cleansing practices have also been evaluated clinically. In a comparative study of 34 hospitalized children with diarrhea, Mahayaty et al. evaluated perianal cleansing with water versus wet tissue using the Scoring System for Diaper Dermatitis Scale (SSDDS). Diaper dermatitis scores decreased in both groups, with a greater numerical reduction in the water group, but the between-group difference was not statistically significant (*p* = 0.179) [54]. In contrast, Price et al. reported that wipe formulation influenced the number of days with clinically significant irritant diaper dermatitis in a larger prospective study [55]. More recent randomized evidence further supports the relevance of cleansing-product formulation, with mildly acidic, pH-buffering baby wipes associated with better diaper-area skin condition than cotton washcloth and water across two clinical trials [56]. These findings suggest that clinical outcomes may depend not only on the cleansing method itself but also on the formulation of the cleansing product. Overall, interpretation of the evidence for cleansing practices is limited by the small number of comparative studies and by heterogeneity in study design, sample size, cleansing formulations, and outcome assessment; therefore, findings from individual products or cleansing regimens should not be generalized across all cleansing approaches.

### 5.2. Clinical Approaches with Heterogeneous Evidence

Clinical studies evaluating topical human milk for the prevention or management of diaper dermatitis have reported heterogeneous findings across different populations and comparators [57,58,59,60]. Potential mechanisms proposed for topical human milk include antimicrobial and immunomodulatory effects, as well as factors involved in tissue repair; however, these mechanisms remain incompletely established in diaper dermatitis. In addition, application of a biological fluid to inflamed or barrier-disrupted skin warrants consideration of hygienic collection, storage, handling, and potential microbial contamination. In a NICU pre–post study of 56 infants, diaper dermatitis occurred in 25% of infants receiving topical human milk compared with 53% in the preintervention group [57]. A randomized three-group study of 48 infants reported improvement in diaper dermatitis severity with topical breast milk [58]. In a randomized trial of 42 infants with established diaper dermatitis, breast milk plus zinc oxide resulted in complete recovery in 61.9% of infants compared with 66.6% with zinc oxide alone, although lesion resolution was faster (2.52 ± 0.67 vs. 3.28 ± 1.14 days; *p* = 0.012) [59]. Another randomized trial of 60 infants comparing breast milk with *Hamamelis virginiana* reported greater improvement with breast milk, although baseline severity differed between groups [60]. Overall, differences in study design, population, comparator, baseline severity, and outcome assessment limit direct comparison across studies and do not establish the superiority of topical human milk over standard diaper-care approaches.

Clinical findings for botanical and natural topical interventions vary by preparation. Colloidal oatmeal was associated with reduced diaper-rash severity in preterm neonates [61]. In a single-blind, three-arm randomized controlled trial of 93 infants aged 1–6 months with mild diaper dermatitis, a barrier cream containing propolis, beeswax, queen bee larvae, and vegetable oil was compared with zinc oxide cream and olive oil using the Assessing the Severity of Uncomplicated Diaper Dermatitis in Infants (ASUDDI) scale. The bee-product formulation was associated with lower diaper dermatitis severity scores during follow-up, particularly on day 2, compared with zinc oxide cream and olive oil [62]. In another clinical study, *Argania spinosa* oil was associated with reduced severity and faster improvement compared with 1% hydrocortisone [63]. In contrast, coconut oil did not show a preventive benefit compared with standard care [39]. An open-label randomized controlled trial involving 60 children aged 1 month to 3 years compared Gojihwa Danti ointment with Siloderm mixi ointment for 7 days using clinical scores for discharge, itching, skin color, and papules. Although Gojihwa Danti ointment showed greater improvement in discharge and itching, complete cure occurred in 80% of the intervention group and 93.3% of the comparator group [64]. Overall, the available evidence should be interpreted separately for each botanical formulation. Findings from individual oils, extracts, or multicomponent preparations cannot be extrapolated to other botanical products because their composition, formulation, comparator, and clinical context differ substantially.

Clinical findings for barrier and targeted topical therapies were formulation- and condition-specific. Clinical evaluation of a novel topical treatment has also reported favorable outcomes in diaper dermatitis; however, these findings remain specific to the formulation evaluated and should not be extrapolated to other topical preparations [65]. A zinc oxide and tocopherol-containing preparation showed greater improvement in selected outcomes [66], and itraconazole-loaded aspasomes showed favorable effects in candidal diaper dermatitis [67], whereas a topical protease inhibitor was not superior to panthenol [68]. Importantly, findings from candidal diaper dermatitis should not be extrapolated to uncomplicated irritant diaper dermatitis.

Clinical studies of non-pharmacological and care-bundle interventions have reported shorter recovery time with more frequent diaper-free air exposure [69]. In a randomized controlled trial of 60 newborns, oxygen was applied locally to the diaper-dermatitis area rather than administered systemically, at 21% FiO_2_ and 5 L/min for 1 h after each diaper change, in addition to routine care [70]. Favorable outcomes have also been reported with a structured multicomponent care bundle [71]. However, these interventions differ substantially in their content and clinical setting, and the contribution of individual components within the care bundle cannot be determined separately.

Maternal education, discharge planning, and transitional care interventions have been associated with a reduced occurrence of diaper dermatitis or diaper rash in clinical studies [72,73,74]. However, diaper dermatitis was often one of several outcomes assessed within broader maternal–infant care programs, and the effects of individual educational or care components cannot be determined separately.

Clinical studies evaluating diaper design have reported product-specific effects on diaper-area skin. Reduced erythema was observed with a shea butter-based emollient diaper compared with a non-emollient diaper [14], while improved diaper-rash-related clinical scores were reported with a high air-through soft diaper compared with a standard diaper [75]. A super-absorbent diaper also showed favorable outcomes in infants with mild-to-moderate diaper rash [76]. These findings suggest that diaper construction, breathability, absorbency, and incorporated emollient systems may influence diaper-area skin outcomes; however, findings from individual products should not be generalized across diaper technologies.

Clinical studies of multicomponent diaper-care regimens have reported improvements in skin pH, erythema, and residual enzymatic activity with the combined use of an emollient-containing diaper and a pH-buffered wipe [30,31]. Because the diaper and wipe were evaluated as part of a combined regimen, the independent contribution of each component cannot be determined. The evidence for these diaper- and wipe-related strategies should also be interpreted in light of the concentration of manufacturer-funded or manufacturer-affiliated studies in this area, underscoring the need for independent replication of the reported findings.

Interpretation of these findings should account for differences in the populations and clinical settings summarized in Table 2; evidence derived from preterm, hospitalized, or other selected clinical populations should not be directly extrapolated to healthy term infants.

Current clinical recommendations advocate a multifaceted preventive approach that includes frequent diaper changes, gentle cleansing, avoidance of irritants, and the use of barrier-supportive products when appropriate [1,2,3,18,24]. Evidence from neonatal care settings further supports a structured approach to diaper-area skin care. Protocol- and quality-improvement-based initiatives have incorporated standardized assessment, perineal care, cleansing, and barrier practices across NICU and other high-risk neonatal populations [5,77,78,79,80].

These findings suggest a potential benefit of care standardization, although implementation-focused evidence should be distinguished from randomized evaluations of individual interventions. In addition, a recent randomized trial in antibiotic-treated term neonates found that oral *Limosilactobacillus reuteri* DSM 17938 did not significantly reduce the incidence of diaper dermatitis compared with placebo [81]. However, translation of these recommendations into routine practice remains inconsistent. Variability in caregiver education, adherence to diaper-care routines, and the continued use of traditional practices may influence clinical outcomes, highlighting the importance of caregiver education and evidence-based preventive strategies.

Several methodological factors contribute to heterogeneity across studies. Variability in clinical assessments and severity-scoring systems for diaper dermatitis, as well as differences in outcome definitions and measurement tools, complicates cross-study comparisons. Differences in the clinical features assessed, scoring ranges, severity thresholds, and definitions of improvement or resolution may result in the same clinical change being classified differently across studies. Consequently, apparent differences in intervention efficacy may partly reflect measurement heterogeneity rather than true differences in treatment effect, limiting direct comparison of effect estimates across studies [16]. Variations in topical formulations and intervention protocols, including different barrier preparations and diapering regimens, hinder direct comparison of clinical outcomes and make it difficult to identify the most effective strategies [1,2,24]. In practice, clinical outcomes depend not only on topical formulations but also on daily diaper-care routines and nursing practices; structured care frameworks and standardized skin-care regimens have been proposed to improve prevention and management of diaper dermatitis in real-world settings [5,69].

Caregiver education is an important component of implementing preventive diaper-care practices consistently [1,2,3].

### 5.3. Emerging and Experimental Strategies

Frequent diaper changes, gentle cleansing, and appropriate barrier protection remain the foundation of first-line diaper care. Although microbiome-targeted approaches are being investigated as potential complementary strategies, direct clinical evidence supporting their use in infant diaper dermatitis remains limited. Accordingly, microbiome-based products should not be considered replacements for established preventive and supportive care measures. Further well-designed clinical studies are needed to determine their safety, efficacy, and potential role in the prevention and management of diaper dermatitis [12,15,46,47,48,50,51].

A first-in-infant clinical trial of a topical postbiotic intervention should evaluate a well-characterized formulation in a randomized design, with comparisons against both the formulation vehicle and standard diaper care where feasible. Randomization or prespecified analyses should account for age and gestational age, particularly given developmental differences in skin barrier function. Outcomes should include a validated diaper dermatitis severity measure together with objective measures such as skin pH and TEWL. If microbiome sampling is incorporated, the protocol should include appropriate negative controls and quantitative microbial endpoints rather than relying solely on relative abundance. Formulation-specific safety and local tolerability should be systematically assessed, and the trial should be prospectively registered before participant enrollment.

## 6. Conclusions

Diaper dermatitis remains one of the most common inflammatory skin conditions in infancy and is associated with barrier disruption, moisture, friction, and changes in the local skin microenvironment. Current evidence supports barrier-protective approaches as an important component of diaper-area care, while findings for pH-related strategies should be interpreted in the context of the specific formulations, study populations, and outcomes evaluated.

At the same time, interest in microbiome-related approaches has grown considerably in recent years. Although microbiome-targeted approaches represent an emerging area of research, current clinical evidence in infants remains limited and is insufficient to support their routine clinical use. Their efficacy, safety, and clinical relevance should be established in appropriately designed infant clinical trials before broader recommendations can be made.

Overall, effective diaper care likely depends on a combination of appropriate cleansing practices, barrier protection, formulation design, and caregiver education rather than a single intervention alone. Future high-quality infant clinical trials using standardized outcome measures will be essential to define the role of barrier-supportive, botanical, and microbiome-oriented interventions in evidence-based diaper care.

## Figures and Tables

**Table 1:** Primary studies characterizing the infant diaper-area skin microbiome.

Study	Population & Clinical Context	Sampling	Microbial Analysis	Main Findings	Major Limitation
Teufel et al. (2021) [13]	18 diaper-wearing infants, 5–15 months; 4-week longitudinal surveillance including healthy and rash-affected skin.	Swabs from genital, intertriginous/leg-fold, perianal, and buttock sites.	Bacterial 16S rRNA (V1–V3) and fungal ITS sequencing; 454 platform; QIIME 1.9 with Greengenes/UNITE.	Microbial composition varied by anatomical site and rash severity. *Staphylococcus* decreased with increasing rash severity, whereas *Enterococcus* and *Candida* increased. Alpha diversity was not significantly associated with rash severity, but beta diversity differed.	Small sample (*n* = 18), limited species-level resolution, and observational design preclude causal inference.
Younge et al. (2024) [43]	46 medically stable preterm infants < 33 weeks’ gestation; randomized to diaper changes every 3 h (*n* = 20) or 6 h (*n* = 26).	Longitudinal buttock swabs before cleansing, collected up to three times weekly; chest and stool samples were also obtained.	16S rRNA sequencing (MiSeq); QIIME 2/DADA2.	Buttock alpha diversity increased over time with no significant difference between diaper-change groups. Change frequency explained only a small proportion of community variation (~4.3%). Higher skin scores were associated with slightly lower diversity, and diversity was inversely related to skin pH.	16S sequencing limits strain-level and functional inference; no a priori microbiome sample-size calculation; preterm population limits generalizability.
Hu et al. (2025) [23]	158 diaper-wearing infants from China, Germany, and the USA; multinational cross-sectional analysis.	D-Squame tape samples from buttocks, perianal region, and thigh; buttock/perianal samples available for 158 infants and thigh samples for 70.	Shotgun metagenomic sequencing (NextSeq 500); MetaPhlAn 4/Kraken 2 and HUMAnN 3.	Microbiome composition differed by anatomical site and geography. *Bifidobacterium longum* on the buttocks was negatively associated with rash, while several taxa showed site-specific positive associations with skin pH or rash severity; functional pathway differences were also reported.	Cross-sectional design prevents causal inference; DNA yield varied by site, and anatomical/geographic heterogeneity complicates interpretation.

**Table 2:** Summary of Clinical Studies Evaluating Preventive and Therapeutic Approaches for Diaper Dermatitis.

Study	Design, Population & Setting	Clinical Purpose	Intervention	Comparator	Follow-Up	Outcome/Instrument	Main Findings	Funding & Main Limitation
O’Connor et al., 2025 [14]	Randomized, double-blinded 2-period, 3-product study; *n* = 271 healthy infants (size-4 diapers); Multicenter/infant wear study	Prevention/diaper-area skin protection	Shea-butter emollient diaper (after 3-wk petrolatum-based emollient run-in)	Non-emollient diaper; continued petrolatum-based emollient diaper	6 wk (3-wk run-in + 3-wk randomized phase)	7-point erythema scale at genital, intertriginous, buttock and perianal sites	Switch to non-emollient increased erythema ≥ 1.5: genital + 11.6% (*p* < 0.05), perianal + 16.5% (*p* < 0.001), all sites + 13.2% (*p* < 0.001); SBE broadly similar to PBE. Safety: No candidal infections noted; well tolerated.	P&G funded; employee authors. Healthy infants, moderate DD excluded; industry-funded; randomized comparison only 3 wk.
Gustin et al., 2020 [30]	Randomized, single-blind, 2-regimen crossover; 61 enrolled, 38 analyzed; age > 7 d to <2 mo; Infant in-use study	Diaper-area skin-barrier outcome	Emollient/apertured diaper + acidic pH-buffered wipe	Non-emollient diaper + limited-buffering wipe	3-d in-use crossover with washout	Skin pH; residual protease activity	Genital pH 5.35 ± 0.08 vs. 5.57 ± 0.09 (*p* < 0.05); residual protease activity 40% lower (*p* < 0.05). Safety: No treatment-related AEs (Adverse events); 6 DD instances without pattern.	P&G funded; employee authors. Very short follow-up; combined regimen; mechanistic/barrier endpoints.
Gustin et al., 2021 [31]	Randomized, investigator-/analysis-blinded 2-regimen crossover; 95 randomized, 80 analyzed; mean age 5.5 wk; Newborn study	Prevention/erythema reduction	Emollient/apertured diaper + acidic buffered wipe	Non-emollient/non-apertured diaper + limited-buffering wipe	Two 14-d periods; 3-d washout before each	7-point erythema scale; skin pH	Perianal erythema reduction 52% vs. 35%; mean 1.01 ± 0.04 vs. 1.12 ± 0.04 (*p* < 0.05); perianal pH 4.94 ± 0.05 vs. 5.28 ± 0.05 (*p* < 0.001). Safety: 12 AEs; none serious/product-related.	P&G funded. Moderate/severe baseline erythema excluded; combined regimen; newborn-only.
Salsbury et al., 2024 [39]	Prospective pragmatic parallel RCT; *n* = 149 preterm/near-term infants (75 vs. 74); median GA 35 wk; USA; Level II NICU, Davenport, Iowa	Prevention	Organic unrefined coconut oil to perineal area at each diaper change	Minimal standard care	During NICU study period	DD log adapted from Minnich severity scale; time to rash/days free of DD	No statistically significant DD-prevention benefit. Safety: AEs documented in 29%; bleeding included as safety endpoint.	Genesis Medical Center personnel/material support; no competing interests. Single-center, open-label/pragmatic; nurse/caregiver-recorded outcomes.
Mahayaty et al., 2021 [54]	Evidence-based nursing implementation, nonrandomized two-group study; *n* = 34 children <5 y with diarrhea (17/17); Indonesia; pediatric infection ward	Prevention/management	Water + soft/cotton cloth perianal cleansing	Wet tissue/wipes	Days 2–4	Scoring System for Diaper Dermatitis Scale (SSDDS)	DD scores decreased numerically with water cleansing, but between-group difference was not significant (*p* = 0.179)	Self-funded; COI nil. Tiny nonrandomized sample; heterogeneous <5-y population; short follow-up.
Price et al., 2021 [55] (BaSICS)	Prospective experimental 3-arm block-randomized study; 722 eligible, 698 completed; birth–8 wk; UK; Greater Manchester, community/home	Prevention/cleansing	Three commercially available wipe formulations; same disposable diaper	Between-brand wipe comparison	55 d	Daily maternal smartphone observations; 5-point IDD scale; clinically significant IDD ≥ 3	Significant IDD ≥ 1 d: 24.6% overall. Multivariable Brand1 vs. Brand3 IRR 1.22 (0.93–1.60), *p* = 0.152; Brand2 vs. Brand3 IRR 1.70 (1.31–2.22), *p* < 0.001. Safety: Severe IDD 2.4%; no allergic reactions requiring medical attention.	Irish Breeze funded; PI paid for writing participation; data owned by funder. Unblinded participants, maternal outcome assessment, imperfect adherence.
Stoltman et al., 2025 [57]	Pre–post quasi-experimental; *n* = 56 (>34 wk GA): human-milk group 24, preintervention 32; USA; Level III NICU	Prevention	Prophylactic topical maternal human milk to diaper area	Preintervention care	NICU period	DD occurrence	DD: 25% (6/24) vs. 53% (17/32).	Funding/COI NR. Nonrandomized; baseline GA/birthweight/LOS imbalance; small single-center sample.
Başkale & Çelik, 2024 [58]	Pretest–posttest experimental study with randomized 3-group allocation; *n* = 48 children 6–18 mo; Türkiye; rural community/FHCs, seven villages	Prevention	Topical breast milk OR maternal diaper-area care education	Control/usual care	2 and 10 wk post-intervention.	Severity of Uncomplicated Diaper Dermatitis in Infants scale	Significant between-group difference favoring topical breast milk (*p* = 0.002)	No specific grant; no competing interests. Small rural sample; *n* = 16/group; limited generalizability.
Veysizadeh et al., 2026 [59]	Randomized clinical trial; *n* = 42 infants < 1 y with clinically diagnosed DD; Iran; Bahrami Children’s Hospital, Tehran	Treatment	25% zinc oxide + topical breast milk	25% zinc oxide alone	7 d	Daily Buckley Diaper Dermatitis Severity Scale	Complete recovery 61.9% vs. 66.6%; lesion resolution 2.52 ± 0.67 vs. 3.28 ± 1.14 d (*p* = 0.012). Safety: Described as safe.	Funding/COI: NR. Small single-center trial; short follow-up; complete recovery not higher with breast milk.
Sevimli Güler et al., 2026 [60]	Randomized single-blind trial; *n* = 60 infants 0–6 mo with DD; Türkiye	Treatment	Topical breast milk	*Hamamelis virginiana* cream (12 mg/100 g)	NR	Validated uncomplicated-DD severity scale	DD score decreased from 5.17 ± 0.46 to 0.03 ± 0.18 with breast milk and from 2.83 ± 1.37 to 1.53 ± 1.11 with Hamamelis cream; between-group *p* < 0.001	Funding/COI: NR. Major baseline severity imbalance limits direct efficacy comparison.
Saleh et al., 2025 [61]	Blinded 3-arm parallel RCT; *n* = 90 preterm neonates with DD (30/group); Egypt; NICU, El-Raml Children’s Hospital, Alexandria	Treatment	Colloidal oatmeal cream	Cold cream; usual NICU care	Daily assessment for 4 d	Buckley Diaper Dermatitis Scale (0–6)	Day 4: 0.00 ± 0.00 vs. 1.50 ± 1.22 cold cream vs. 1.97 ± 1.33 usual care; oatmeal vs. both *p* < 0.001, d > 0.8	No specific grant; no competing interests. Small single-center preterm sample; short primary assessment.
Aksoy et al., 2026 [62]	Single-blind, three-arm randomized controlled trial; *n* = 93 (31/group); infants aged 1–6 mo with mild DD; Türkiye; 3 FHCs + pediatric outpatient clinic	Treatment	Barrier cream with propolis + beeswax + queen-bee larvae + vegetable oil	Zinc oxide barrier cream; olive oil	3 d	Assessing the Severity of Uncomplicated Diaper Dermatitis in Infants (ASUDDI) scale; days 1–3	Barrier cream 1 lower severity; day-2 difference *p* = 0.012; vs. olive oil reported as 2.54-times more effective (*p* = 0.004)	Funding/COI NR. Mild DD only; exact *n*/effect metric/scale not available in abstract.
Alsatari et al., 2023 [63]	Quasi-experimental study with randomized allocation; 147 randomized, 140 completed (70/group); age 1–24 mo; Jordan; 3 hospitals + 3 primary-care centers, Irbid	Treatment	Cold-pressed *Argania spinosa* oil 4×/day	Product described by authors as 1% hydrocortisone ointment	7 d	Diaper Dermatitis Grading Scale (0–4)	Day-3 complete healing 67.1% vs. 45.7%; adjusted odds of higher DD grade OR 0.246 (95% CI 0.072–0.842), *p* = 0.025. Safety: No allergic reactions/adverse effects reported.	Jordan Univ. of Science & Technology grant; no COI. Regional study; diaper type uncontrolled; comparator-description inconsistency in article.
Momin et al., 2025 [64]	Open-label, two-arm parallel randomized controlled trial; *n* = 60 children aged 1 mo–3 y.	Treatment	Gojihwa Danti ointment	Siloderm mixi ointment	7 d	Clinical assessment of discharge, itching, skin color, and papules	Both groups improved; Gojihwa Danti showed greater improvement in discharge and itching. Complete cure: 80% vs. 93.3% with comparator.	Funding/COI: NR.
Mirle et al., 2024 [66]	Prospective randomized interventional study; *n* = 88 infants < 12 mo with newly diagnosed DD; India; tertiary-care teaching hospital, Mangalore	Treatment	10% zinc oxide + tocopherol 3×/day	10% zinc oxide 3×/day	5 d	Buckley DD Severity Score; rash dimensions; skin pH	Day-5 DDSS 1.96 ± 0.77 vs. 2.72 ± 1.27 (*p* = 0.001); DDSS reduction 2.91 ± 0.73 vs. 1.84 ± 0.72 (*p* < 0.001). Safety: No side effects noted; AEs not formally assessed.	No specific grant; no COI. Not blinded; time-to-healing not recorded; one tocopherol dose.
Lamie et al., 2022 [67]	Randomized parallel double-blinded formulation study; DD-relevant candidal subgroup *n* = 60 infants (6 groups of 10), age 5–25 mo; Egypt; Minia University Hospital dermatology outpatient clinic	Treatment—candidal DD	Itraconazole aspasomal creams (0.1%/0.5%) and related formulations	Non-formulated ITZ, plain aspasome and cream base arms	10 d	Rash area/redness/pustules; dermoscopy; *Candida* skin scrapings	0.5% aspasomal ITZ: rash area 47.1 ± 8.6 → 0 cm^2^; redness/pustules and mycology 10/10 positive → 0/10 Safety: Parents reported no adverse effects.	Funding/COI: NR. *n* = 10/arm; specialized candidal DD; multiple experimental formulations; not generalizable to uncomplicated irritant DD.
Huijgen et al., 2025 [68]	Single-center double-blind RCT; 30 randomized, 28 treated, 26 evaluable; diaper-wearing children < 3 y after GI surgery; Netherlands; Erasmus MC/Sophia Children’s Hospital	Prevention	5% potato protease-enzyme-inhibitor cream	5% panthenol cream	Planned 4 wk	Buckley 0–6 scale from standardized perianal photographs	DD 66.7% (8/12) vs. 78.6% (11/14), *p* = 0.665; high early DD caused frequent discontinuation. Safety: One possible perineal candidiasis; no other cream-related complications.	EuroDrug supplied blinded medication; no role reported in conduct/analysis/writing. Small single-center postoperative sample; incomplete 4-wk data.
Bilgiç et al., 2026 [69]	Prospective parallel RCT; 140 randomized, 130 analyzed (66/64); term infants 0–3 mo with mild DD; Türkiye; private hospital pediatric outpatient clinic	Treatment	Diaper-free air 5 min × 12/day (60 min/day)	Diaper-free air 5 min × 6/day (30 min/day); standardized co-care	Until healing; all healed by day 4	UDDSRSI (0–6), daily standardized photographs with blinded raters	Mean healing time 1.98 ± 1.22 vs. 3.23 ± 1.01 d (*p* = 0.002); all healed by day 4.	No funding; no competing interests. Mild DD only; single center; no no-air/usual-care arm; incomplete blinding.
Doğan & Dinç Kaya, 2024 [70]	RCT; *n* = 60 newborns with DD (30/30); Türkiye; single-center NICU	Treatment	Routine diaper care + local oxygen therapy (21% FiO_2_, 5 L/min for 1 h after each diaper change)	Routine diaper care	72 h	DD severity and recovery assessed at 24, 48, and 72 h	Recovery OR 6.46 (95% CI 2.30–18.19) at 24 h; 5.12 (2.16–12.18) at 48 h; 7.98 (reported CI 1.75–8.93) at 72 h. Safety: No observed side effects reported.	TÜSEB grant 22,960; no COI. Small single-center neonatal sample; limited replication.
Chin et al., 2024 [71]	Nonrandomized historical-control cohort; *n* = 54 children 0–24 mo after CHD surgery (30 bundle/24 historical); Taiwan; Taichung PICU	Prevention + management	DD care bundle: ≥q3h changes + dimethicone/sodium-hyaluronate protectant; antifungal if candidiasis	Historical usual cleansing care	During postoperative hospitalization; skin assessed every 8 h	DD development	DD 6.67% vs. 41.65% (*p* = 0.002); OR 0.10 (95% CI 0.019–0.520), *p* = 0.006.	Supported by non-US government funding; exact funder NR. Historical control; small specialized postoperative cohort; bundle effect cannot be decomposed.
Jabraeili et al., 2026 [72]	Parallel randomized clinical trial; *n* = 90 newborns < 28 d, BW > 2000 g, breastfed; baseline DD excluded; Iran; Level II NICU, northwest Iran	Prevention	Comprehensive maternal education + participation in diaper-area care	Routine NICU diaper care	At least 4 d	DD presence, severity and extent.	Lower DD severity/extent with intervention; between-group differences day 2 *p* = 0.01, day 3 *p* < 0.001, day 4 *p* = 0.002.	No funding; no COI. Short follow-up with discharge attrition; multifaceted program prevents attribution to individual components.
Cheng et al., 2023 [73]	Randomized controlled study; maternal–infant teach-back intervention; *n* reported in source as 130/128 groups.	Secondary DD outcom	Routine postpartum education + teach-back	Routine education	42 d postpartum	Infant health outcomes including diaper dermatitis	DD incidence: 14.8% (19/128) vs. 32.3% (42/130); χ^2^ = 10.896, *p* = 0.001.	University of Science and Technology of China Undergraduate Quality Engineering Project (2022xjyxm091); no competing interests.
Tutar et al., 2025 [74] (TRAMPRE)	Randomized controlled study; *n* = 133 mothers of late-preterm infants; tertiary-care NICU, Türkiye.	Secondary DD/post-discharge complication outcome	Transition Model–Based discharge training (TRAMPRE), including infant-care discharge training, Preterm Infant Care Guide, and telephone follow-up/counseling	Usual discharge care	Post-discharge follow-up	Post-discharge complications, including diaper rash	Diaper rash: 4.5% in the intervention group vs. 30.3% in the control group; significant between-group difference.	No specific funding; no conflicts of interest. DD was a secondary outcome within a multifaceted transitional-care intervention; therefore, the effect of individual intervention components cannot be determined.
Salvi/Anjali et al., 2021 [75]	Single-blind randomized controlled crossover; 114 recruited, 105 completed; age 3–24 mo, absent-to-mild rash; India; C.L.A.I.M.S Pvt. Ltd., Mumbai	Prevention/diaper-area skin outcome	High Air-Through soft diaper	Usual/favorite standard disposable diaper	4 wk; 2 wk/product	Study-specific total diaper-rash score across multiple sites	First 2 wk score 5.05 ± 1.97 vs. 6.16 ± 2.22; increase from baseline smaller with HAT (*p* = 0.045); crossover-period change *p* = 0.001. Safety: No other AEs reported.	Kao Corporation funded; 3 coauthors employees. No reported washout; environment uncontrolled; industry-funded.
Diana/Arline et al., 2020 [76]	Single-blind randomized controlled crossover; safety *n* = 113; efficacy *n* = 59 infants 3–24 mo with mild–moderate DD; Indonesia; PT Equilab International, Jakarta	Prevention + treatment	New-technology super-absorbent diaper	Standard absorbent diaper	4 wk; 2 wk/product	Skin Assessment Score (SAS) across 6 diaper regions	Total SAS improvement during super-absorbent periods not significant; pubic-area SAS improved in both sequences (*p* = 0.011 and *p* < 0.049). Safety: No major safety signal reported.	KAO Corporation funded; company-affiliated authors. Small efficacy sample; no washout; TEWL/environment not measured.
Brandon et al., 2022 [52]	Parallel RCT; 48 randomized, 46 analyzed; born ≤ 32 wk GA; intervention started ~35 wk PMA; USA; Level IV NICU, Southeastern academic center	Diaper-area skin-barrier outcome/prevention	Routine diaper changes every 6 h; earlier if stool	Standard 3-h changes	6 wk	Buttock pH, TEWL, Neonatal Skin Condition Score	No significant group differences: buttock pH b = −0.07 *p* = 0.60; TEWL b = −5.26 *p* = 0.31; NSCS OR = 0.79 *p* = 0.76. Safety: DD in 1/46; no intervention safety signal highlighted.	Kimberly-Clark funded; design input/interpretation/editing role. Small single-center stable-preterm sample; staff unblinded; DD not primary endpoint.
Dall’Oglio et al., 2021 [65]	Prospective open-label uncontrolled trial; *n* = 20 total, including 10 children (1 d–4 y) with mild–moderate DD; Italy; University of Catania dermatology clinic	Treatment	Barrier cream: zinc gluconate–taurine complex + zinc oxide + panthenol + glycerin + shea butter	None	30 d	CEA 0–4; IGA; photography; tolerability	Pediatric day-30 IGA: complete 6/10, excellent 2/10, moderate 1/10, mild 1/10; pooled CEA result includes adults. Safety: No signs of local intolerance; tolerability excellent in 90% overall.	No funding; no COI. Uncontrolled, open-label; pediatric *n* = 10; adult/pediatric pooled quantitative results.
Glashan, 2022 [77]	Pre–post implementation/QI study; *n* = 33 (25 preimplementation, 8 postimplementation); infants with NAS, > 36 weeks’ gestation and <3 mo old, NICU.	Prevention	5-component skin-care protocol: DD tool, frequent superabsorbent-diaper changes, petroleum jelly/alcohol-free wipes, pH-neutral bathing, zinc oxide for erythema	Historical/preimplementation care	6-mo preimplementation and 5-mo postimplementation periods	DD incidence assessed using the Scoring System for Diaper Dermatitis Scale (SSDDS; 0–6).	DD decreased from 64% (preimplementation) to 12.5% (postimplementation), *p* ≤ 0.01; reported 80.5% relative decrease	COI none; funding NR (Not reported). Small sample; markedly unequal groups (25 vs. 8); single NICU; pre–post nonrandomized design; possible undocumented diaper changes/emollient applications.
Sharp, 2025 [78]	Uncontrolled pre–post QI project; *n* = 164 neonates (89 pre/75 post); USA; Oklahoma City, Level IV NICU	Prevention + management	DD scoring tool embedded in prevention/management algorithm + staff education + bedside/EMR implementation	Historical/preimplementation care	3-mo project period	DD occurrence and severity score	No significant pre–post differences in DD occurrence or severity. Safety: NR	Funding/COI: NR. Concurrent introduction of water-based wipes confounded attribution; nonrandomized historical comparison.
Rogers et al., 2021 [79]	Uncontrolled pre–post QI project; *n* = 1070 NICU infants admitted ≥ 1 d; USA; University of Utah Hospital, Level III NICU	Prevention + management	Standardized severity-based perineal skin-care guideline + preservative-free wipes	Historical/preimplementation care	Jul 2017–Mar 2019	4-level pictorial DD classification; incidence/severity; severe-DD duration	Overall DD incidence decreased 16.7%; severe DD 34.9%; severe-DD duration by 3.5 d/100 patient-days. Safety: No increase in fungal skin infection.	COI none; funding NR. Multicomponent QI intervention; nonstandardized baseline documentation; individual component effects cannot be isolated.
Railsback & Karas, 2026 [80]	Pre–post quality-improvement project; high-risk neonates in a Level III NICU; *n* = 54 in the initial trial	Prevention	Cavilon No Sting Barrier on admission, reapplied every 3 d and/or after bathing	Historical/preimplementation data	3-mo initial trial; during NICU stay	Development of DD during hospitalization	DD incidence < 10% among eligible high-risk neonates during the initial phase (*n* = 54), compared with a historical unit rate of 53%.	No specific funding; no relevant financial interests or commercial affiliations disclosed.
O’Connor et al., 2025 [56]—Study 1	Prospective randomized examiner-blinded parallel trial; 110 randomized, 105 completed; age ~3–9 mo; 55/55 randomized; Germany; Hamburg	Diaper-area skin-barrier outcome/prevention	Pampers Aqua Pure wipe (99% water, mildly acidic pH-buffering system)	100% cotton washcloth + water	7-d washout + 14-d use	7-point erythema scale (0–3, 0.5 increments); TEWL; skin pH	Mean perianal erythema 0.12 points lower (*p* = 0.036); genital erythema 0.139 lower (*p* = 0.0013); buttock TEWL day 14 lower by 1.817 g/m^2^/h (*p* = 0.0002). Safety: 67 treatment-emergent AEs in 52 infants; none serious; 6 diaper-rash AEs equally distributed.	P&G funded; multiple P&G-employed authors. Caregivers unblinded; 14-d follow-up; limited racial/skin-type diversity; moderate/severe skin disease excluded.
O’Connor et al., 2025 [56]—Study 2	Prospective randomized examiner-blinded parallel trial; 106 randomized, 103 completed; age up to 36 mo; erythema analysis 51/54; UK; Chelmsford, Essex	Diaper-area skin-barrier outcome/prevention	Pampers Aqua Harmonie wipe (99% water, mildly acidic pH-buffering system)	100% cotton washcloth + water	7-d washout + 14-d use	7-point erythema scale (0–3, 0.5 increments); TEWL; skin pH	Mean perianal erythema 0.10 points lower (*p* = 0.019); no TEWL difference; skin pH lower at measured diaper-area sites. Safety: 27 treatment-emergent AEs in 20 infants (11 wipe/16 WCW); none serious; 4 diaper-rash AEs, 2/group.	P&G funded; multiple P&G-employed authors. Caregivers unblinded; 14-d follow-up; limited racial/skin-type diversity; moderate/severe skin disease excluded.
Majima & Kobayashi, 2022 [53]	Comparative intervention study; *n* = 118 full-term newborns (63 moisturizer/55 control); Japan	Diaper-area skin-barrier outcome/prevention	Perianal moisturizer from day 1 of life	Perianal care without moisturizer	Birth to 1-mo visit	Perianal TEWL, stratum corneum hydration (SCH), skin-surface pH	At 1 mo: TEWL 19.4 vs. 25.8 g/m^2^/h (reported *p* = 0.00); SCH 58.8 vs. 46.5 a.u. (reported *p* = 0.00); pH not significantly different. Safety: NR.	University of Yamanashi + Natural Science Co., Ltd.; company support/equipment. Physiologic barrier endpoints; DD/rash incidence efficacy not established.
Železnik et al., 2026 [81]	Randomized, double-blind, placebo-controlled trial; secondary analysis; 89 term neonates treated with antibiotics in the first 3 wk of life; DD data available for 87; three centers, Slovenia	Secondary DD outcome/prevention	*Limosilactobacillus reuteri* DSM 17938, 1 × 10^8^ CFU/day (5 drops daily), started with antibiotic treatment	Identical placebo without probiotic bacteria	6 wk	DD incidence and onset assessed by parental questionnaire; DD defined as erythematous rash with or without scaling, swelling, papules, or ulcers	DD occurred in 30% of probiotic vs. 25% of placebo infants; no significant difference. Fungal DD occurred in 7% in both groups. No significant effect on gut microbiota composition or diversity.	Specific DD-related safety events: NR. Slovenian Research and Innovation Agency and University Medical Centre Ljubljana support; study products supplied by BioGaia, which authors reported had no role in planning, conduct, analysis, or interpretation. Secondary/exploratory DD analysis; DD assessed by parental questionnaire; limited to antibiotic-treated term neonates and not powered primarily for DD.

## Data Availability

No new data were created or analyzed in this study. Data sharing is not applicable to this article.

## Supplementary Materials

The following supporting information can be downloaded at: https://www.mdpi.com/article/10.3390/microorganisms14102271/s1, Table S1: Database-specific search strategies.

## Abbreviations

The following abbreviations are used in this manuscript:

AE	adverse event
DD	diaper dermatitis
IDD	irritant diaper dermatitis
IL-1α	interleukin-1 alpha
IL-8	interleukin-8
NR	not reported
TNF-α	tumor necrosis factor alpha
TEWL	transepidermal water loss
